# Dynamics of photoexcited 5-bromouracil and 5-bromo-2′-deoxyuridine studied by extreme ultraviolet time-resolved photoelectron spectroscopy in liquid flat jets†

**DOI:** 10.1039/d4sc03920c

**Published:** 2024-09-26

**Authors:** Do Hyung Kang, Masafumi Koga, Neal Haldar, Daniel M. Neumark

**Affiliations:** a Department of Chemistry, University of California, Berkeley California 94720 USA; b Chemical Science Division, Lawrence Berkeley National Laboratory Berkeley California 94720 USA dneumark@berkeley.edu

## Abstract

The UV-induced photo-relaxation dynamics of 5-bromouracil (BrU) and 5-bromo-2′-deoxyuridine (BrUrd) in aqueous solution were investigated using femtosecond time-resolved photoelectron spectroscopy with an extreme ultraviolet (XUV) probe in a flat liquid jet. Upon excitation to the ^1^ππ* state by 4.66 eV UV photons, both molecules exhibited rapid relaxation into lower-lying electronic states followed by decay to the S_0_ ground state. By employing a 21.7 eV XUV probe pulse, we were able to differentiate the relaxation of the excited state population from the initially excited ^1^ππ* state to an intermediate electronic state with 100 fs. Computational results identify this intermediate as the ^1^πσ* excited state, accessed by a ^1^ππ*/^1^πσ* conical intersection, and the signal from this intermediate state disappears within ∼200 fs. In contrast to thymine, formation of neither the ^1^nπ* state nor a long-lived triplet state was observed. Although the ^1^πσ* state is largely repulsive, prior studies have reported a low quantum yield for dissociation, and we observe weak signals that are consistent with production of hot S_0_ ground state (for BrUrd) on a time scale of 1.5–2 ps. It thus appears that solvent caging effects limit the dissociation yield in solution.

## Abstract 2

The UV-induced photo-relaxation dynamics of 5-bromouracil and 5-bromo-2′-deoxyuridine in aqueous solution were investigated using femtosecond time-resolved photoelectron spectroscopy with an extreme ultraviolet probe in a flat liquid jet.

## Graphical abstract

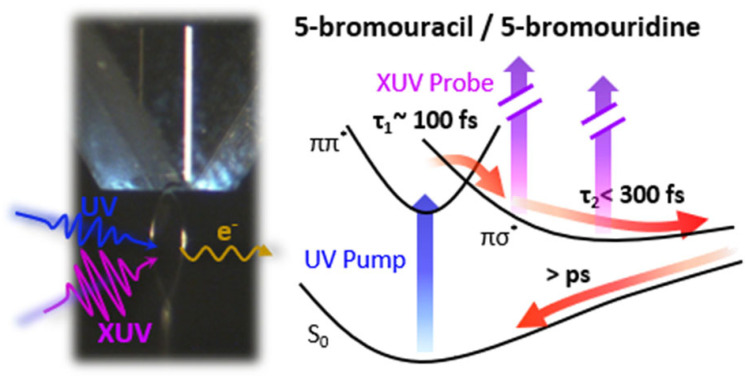

## 1. Introduction

**Scheme 1:**
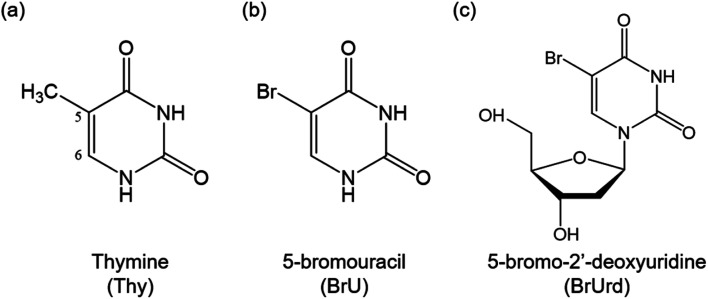
Molecular structures of (a) thymine (Thy), (b) 5-bromouracil (BrU), and (c) 5-bromo-2′-deoxyuridine (BrUrd).

UV irradiation of nucleic acid constituents (NACs) triggers photochemical reactions in DNA bases, potentially leading to serious damage to both the NACs and adjacent biological tissues.^1–3^ However, the extent of DNA damage is known to be mitigated by the efficient relaxation of nucleobases to their electronic ground state, rapidly dissipating the excess energy into the surroundings.^4–6^ Modification of this pathway is of considerable interest in the development of highly effective radiosensitizers for treating various types of cancers. Upon absorption of UV photons or attachment of an excess electron, radiosensitizers generate highly reactive photoproducts that damage or destroy the cancer cell.^7,8^ Halogenated uracils have been widely recognized as representative radiosensitizers, sharing structural analogies with thymine (Thy, Scheme 1), and are commonly substituted for Thy in DNA strands.^9–14^ This substitution does not significantly alter biological structure but exhibits a potent radiosensitizing effect. Despite the considerable importance of halogenated uracils, a fundamental understanding of photochemical processes in these molecules remains incomplete. Notably, the influence of solvation on the excited state relaxation process has been relatively overlooked, despite its anticipated significant impact on relaxation dynamics compared to those in an isolated environment.

In this study, we investigate the photo-relaxation dynamics of 5-bromouracil and 5-bromo-2′-deoxyuridine (BrU and BrUrd, Scheme 1), two prototypical halogenated uracil radiosensitizers, in aqueous solution by means of extreme ultraviolet (XUV) time-resolved photoelectron spectroscopy. Previous theoretical investigations on gas-phase BrU proposed that UV photoexcitation to the first bright ^1^ππ* state is followed primarily by rapid decay to the electronic ground state (S_0_) through a ^1^ππ*/S_0_ conical intersection (CI).^15,16^ Alternatively, C–Br dissociation *via* nonadiabatic coupling to the optically dark ^1^πσ* state is feasible. Both pathways involve substantial geometric deformation of the Br atom out of the pyrimidine ring plane and must overcome a potential energy barrier of ∼0.2 eV.^16^ Additionally, relaxation to the optically dark ^1^nπ* state is conceivable, as is observed in the relaxation dynamics of other natural DNA bases.^17,18^ The formation of the ^1^nπ* state and subsequent intersystem crossing (ISC) to the triplet ^3^ππ* state may trap the reaction flux for longer times, but the role of these states remains controversial.

In aqueous solution, the dynamics become less distinct due to interactions with solvent networks altering the energetic location of excited states. In general, hydrogen bonding stabilizes ππ* states but substantially destabilizes states with nπ* character, potentially altering the barrier height to nonadiabatic coupling to the pathways that produce specific photoproducts.^19,20^ As these electronically excited states lie in close proximity in the UV excitation region, understanding solution-phase dynamics is crucial to discern whether BrU acts as a radiosensitizer through its own photochemical reaction or requires external electron engagement (*e.g.*, pre-hydrated electron transfer) to trigger dissociative electron attachment (DEA) on its anionic states.^21,22^

Previous femtosecond transient absorption (TA) spectroscopy revealed rapid decay of the bright ^1^ππ* state with a lifetime of ∼0.4 ps in aqueous BrUrd upon 266 nm excitation,^23,24^ but the detailed relaxation pathway remained elusive. More recently, time-resolved broadband TA spectroscopy, in conjunction with steady-state absorption and fluorescence spectroscopies, suggested formation of the ^1^nπ* state within ∼1 ps, followed by relaxation to the S_0_ ground state in over 400 ps *via* a planar ^1^nπ*/S_0_ CI.^25^ Despite these experimental findings, discrepancies persist between experimental and theoretical studies of the relaxation dynamics of BrU and BrUd, particularly regarding the involvement of the ^1^πσ* state that can lead to C–Br bond dissociation. These ambiguities in the BrU relaxation pathway underscore the need for more definitive experimental techniques capable of clearly distinguishing between electronic states.

Motivated by these considerations, we investigate the photophysics of BrU and BrUrd in aqueous solution by femtosecond time-resolved photoelectron spectroscopy of a flat liquid jet using an XUV probe pulse. The advantages of XUV probe pulses over lower energy UV probes lie in the ability to differentiate electronic states involved in the overall relaxation pathway by electron binding energy (eBE) and to track their temporal behavior in a time-resolved manner with extended probing energy windows.^26–28^ In our study, we identify two decaying components in BrU and BrUrd in aqueous solution, with lifetimes of 77 fs and 171 fs for BrU, and 104 fs and 268 fs for BrUrd. These lifetimes are attributed to relaxation *via* the ^1^πσ* state, with possible subsequent production of the S_0_ ground state. We find no evidence of a nonadiabatic transition to the ^1^nπ* state nor for ISC to form a triplet state.

## 2. Methods

Our implementation of XUV liquid flat jet photoelectron spectroscopy (XUV-LJPES) is outlined elsewhere.^29,30^ In brief, a liquid flat jet was generated using a microfluidic chip device (Micronit B.V.) operating in the gas-dynamic mode.^31–33^ This was achieved by introducing the sample solution into a central channel with a diameter of 30 μm, while helium colliding gas was introduced into two side channels, each with a diameter of 50 μm. Gas from each side channel merged with liquid from the central channel at an angle of ∼40° at the end of the chip, forming a gas dynamic flat jet by compressing the cylindrical jet from both sides. The flat jet leaf has a measured width of ∼200 μm and a length of ∼500 μm with an estimated thickness in tens of nm.^31,34^ The flow rates of the sample solution and helium gas were carefully controlled to maximize the size of the flat jet leaf while preventing the spread of a second leaf. In the current experiment, the sample solution was pumped at a flow rate of approximately 0.2 mL min^−1^ using an HPLC pump (Shimadzu, LC-40i), and the pressure of the helium colliding gas upstream of the chip was set to approximately 300 kPa. As described previously,^30^ the flat jet yields higher pump-probe signals owing to better spatial overlap with the pump and probe light pulses. Moreover, when operated in the gas dynamic mode, photoelectron signal from the liquid is enhanced compared to that from the water vapor jacket surround the liquid jet. Finally, the time-dependent space charge effect^35^ is lower for the gas dynamic flat jet, an observation that will be described in more detail in an upcoming publication.^36^

Solutions comprised either 8 mM of BrU (Sigma-Aldrich, 98%) or 15 mM of BrUrd (TCI chemicals Inc., >98% HPLC grade) in 4 mM Trizma HCl buffer solution at pH ∼8 without further purification; the lower solubility of BrU necessitates a more dilute solution. To mitigate effects from streaming potentials and jet charging,^37–39^ 25 mM NaCl was added to the solution. The flat jet was positioned ∼1 mm away from a 450 μm skimmer orifice, and its position was finely adjusted using a set of XYZ-piezo linear actuators (Newport, 8301NF) to maximize the photoelectron signal from the solution. The incidence angle of the UV/XUV pulse onto the flat jet face was set at ∼60° to minimize UV laser scattering signal in the photoelectron spectrum. After passing through the laser interaction region, the sample solution was collected and frozen by a liquid nitrogen trap.

Photoelectron spectra were recorded using a magnetic-bottle time-of-flight (TOF) photoelectron spectrometer.^40^ In the laser interaction region, a stack of neodymium permanent magnets, capped with a soft iron cone, generated a strong magnetic field (∼0.6 T at the cone tip and ∼0.4 T at the laser interaction region) that guided photoelectrons generated from the liquid jet into the skimmer orifice. Subsequently, a 66 cm long solenoid tube, maintaining an 8 G homogeneous magnetic field, converted the longitudinal motion of the electrons into axial motion, directing them into a chevron-type microchannel plate (MCP) detector coupled to a phosphor screen. Photoelectron signals were taken from the phosphor screen and were amplified by a variable preamplifier (Stanford Research Systems, SR446). The TOF distribution of the photoelectrons was recorded by an ADC digitizer card (Acqiris, U5309A) and Labview-based DAQ software.

Femtosecond laser pulses were generated by a 1 kHz repetition femtosecond Ti:sapphire regenerative amplifier (Coherent, Astrella USP) seeded by a femtosecond Ti:sapphire oscillator (Coherent, Vitara-S). A 797 nm-centered (*ω*) 7 mJ femtosecond fundamental output (35 fs in FWHM) was split using a series of beam splitters. 1 mJ of the fundamental output was used to generate the frequency-tripled 266 nm (3*ω*) UV pump pulse using two beta-barium borate (BBO) crystals. This UV pump pulse was focused and introduced into the liquid flat jet by an annular concave mirror with a 2 m long focal length. Another 5 mJ of fundamental output was frequency-doubled (2*ω*) by a 200 μm thick BBO crystal to drive high-harmonic generation (HHG). A convex lens with a 1 m focal length focused the 2*ω* HHG driver (at 400 nm) into a semi-infinite gas cell (SIGC) filled with ∼3 torr of Kr gas,^41,42^ after which the 400 nm light was filtered out by a 200 nm thick Al filter. XUV light at the 7th harmonic of the 400 nm driving pulse (14*ω*, 21.7 eV) was reflected by a toroidal mirror and then a multilayer mirror, which served to focus the XUV pulse onto the liquid jet and select the 7th harmonic. Residual 9th harmonic in the XUV pulse was additionally filtered out by a 300 nm thick Sn filter (Lebow Company) between the mirror and liquid jet chamber. The UV pump-XUV probe delays were controlled and scanned by a linear translational stage (Newport, DL225).

## 3. Results and analysis

**Fig. 1:**
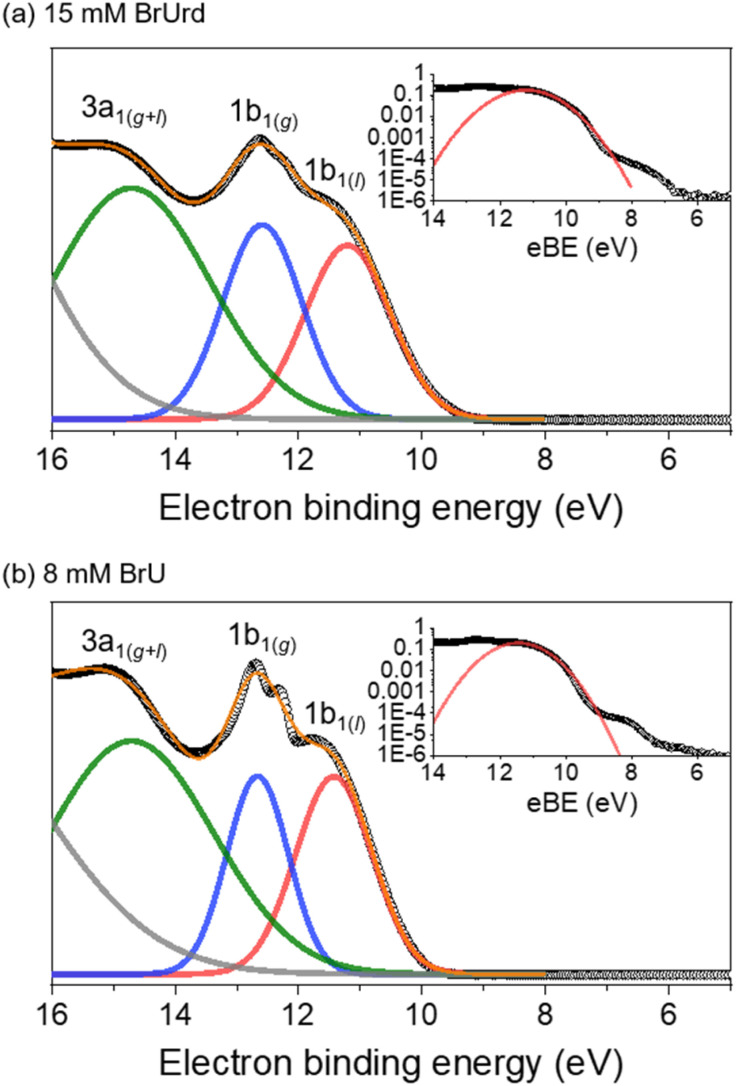
XUV (21.7 eV)-only photoelectron spectra of (a) 15 mM BrUrd and (b) 8 mM BrU aqueous solutions in liquid flat jets. Experimental data points are shown in open circles. Each spectrum was fitted with four Gaussian functions representing (gray) 1b_2(g+l),_ (green) 3a_1(g+l)_, (blue) 1b_1(g)_, and (red) 1b_1(l)_ of water. Orange solid line represents total fit of three Gaussian functions. Insets show logarithmically scaled plots with a Gaussian fit representing 1b_1(l)_ signal in red solid line. Weak features at lower eBE than the 1b_1(l)_ peak indicate photoelectron signals from the target solute molecules.

Fig. 1 shows one-color XUV (21.7 eV) photoelectron spectra for 15 mM BrUrd and 8 mM BrU aqueous solutions in liquid flat jets. A deconvoluted fit with four Gaussian functions identifies the contributions from gas and liquid water photoionization.^28,43^ The peaks at eBEs of 12.6 eV and 14.7 eV correspond to the water vapor 1b_1(g)_ and 3a_1(g)_ (and 3a_1(l)_) peaks, respectively, which originate from the ionization of vapor-phase H_2_O molecules near the surface of the flat jet. The fit for the low eBE side of the 1b_2(g+l)_ peak is also shown (grey), but its position was not precisely determined, varying between 18.0 and 18.5 eV depending on the solution. In the lower eBE region, a broad feature around 11.3 eV eBE is attributed to photoelectrons from the liquid water 1b_1(l)_ peak. The photoelectron signal from both target molecules (BrU and BrUrd) begins to emerge at ∼7 eV as weak broad features adjacent to the 1b_1(l)_ peak. These features are attributed to one-photon ionization of BrUrd or BrU by the XUV pulse. They were not seen in the photoelectron spectrum of a 25 mM NaCl aqueous solution (Fig. S1†). The onsets of the solute features are at ∼6.8 eV for both BrUrd and BrU, but the maximum intensities were difficult to precisely evaluate due to the broad spectral width and some overlap with the water 1b_1(l)_ peak. A previous theoretical study utilizing DFT calculations in aqueous solution predicted the vertical ionization energy (VIE) of BrU to be 6.82 eV,^44^ close to the experimental value obtained in this study.

**Fig. 2:**
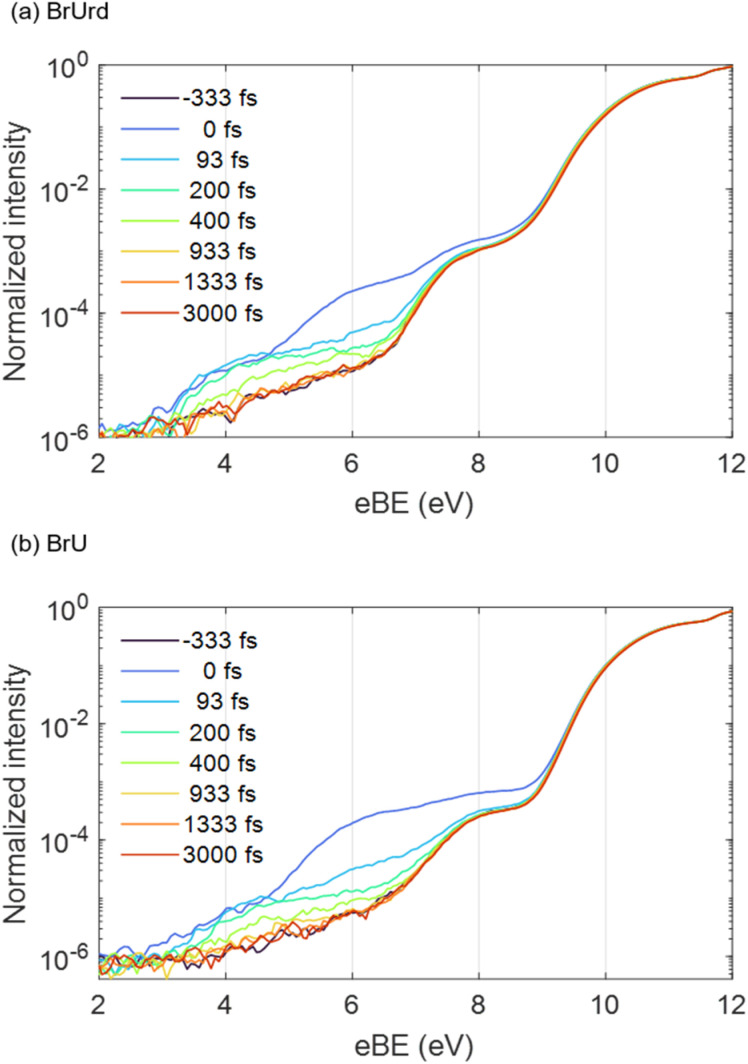
Spectral lineouts of time-resolved photoelectron spectra obtained from (a) 15 mM BrUrd and (b) 8 mM BrU aqueous solutions at different delays between the pump (4.66 eV) and probe (21.7 eV) pulses. The data are normalized to the water 1b_1(l)_ peaks. Note that the *y*-axes are logarithmic.

Time-resolved photoelectron spectra are acquired using 4.66 eV (266 nm) UV pump and 21.7 eV (57 nm) XUV probe pulses while scanning the delays between the pump and probe pulses. The photoelectron spectra at several pump-probe delays are shown in Fig. 2. For BrUrd (Fig. 2a), a significant enhancement in photoelectron signal is observed in the 5–8 eV eBE region at zero-time delay (*t*_0_); this is attributed to the laser-assisted photoelectric effect (LAPE) from the liquid water^45^ that persists only during temporal overlap between the pump and probe pulses and is gone by 100 fs. In addition, signal from 3 to 7 eV is seen that persists up to approximately ∼400 fs. This corresponds to the pump-probe signal representing the initial population of the first ^1^ππ* state and its subsequent decay. For BrU, the pump-probe signal from 3 to 7 eV is slightly less pronounced compared to BrUrd owing to the lower concentration of the BrU solution. We note that modifications in our XUV beamline alignment have yielded about an order of magnitude improvement in our pump-probe signal compared to earlier work.^30^

The presence of space charge effects causes spectral shifts in the photoelectron spectra originating from the liquid phase (both solute and solvent), depending on the pump-probe delays.^28,35^ To evaluate the extent of these space charge shifts, the 1b_1(l)_ and 1b_1(g)_ water peaks are fitted with double-Gaussian functions for each delay, and the delay-dependent space charge shift is evaluated.^28,30^ The delay-dependent spectral shifts of the 1b_1(l)_ peak were then employed to correct the space-charge shift for the overall photoelectron signals from the solutes. Fig. S2† shows the double-Gaussian fitting of the photoelectron signal and the space-charge shift *vs.* delay time. The extent of the shift is influenced by UV (266 nm) absorption cross-section, concentration of the target solute, and UV photon intensity, all of which determine the number of cations generated by multiphoton ionization. The time-resolved data shown below were obtained up to delay times of 4 ps and exhibit a space-charge shift of ∼0.03 eV, which is negligibly small compared to the broad spectral bandwidth (>1 eV) of the excited state signals.

**Fig. 3:**
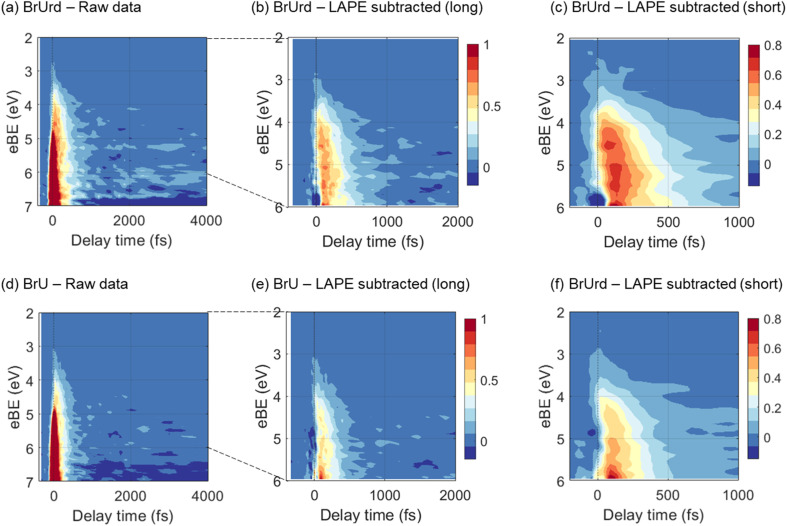
Contour plots of time-resolved photoelectron spectra for (a–c) 15 mM BrUrd and (d–f) 8 mM BrU aqueous solutions in liquid flat jets. The 2–6 eV region of the raw photoelectron spectra (a and c) was processed by subtracting the laser-assisted photoelectric effect (LAPE) at zero-time delay obtained in a 25 mM NaCl aqueous solution, resulting in LAPE-subtracted spectra with long (b and e) and short (c and f) temporal windows. The depletion features emerging from 6.5–7 eV corresponds to the UV-pump-induced population depletion of the target molecule ground state. 3 point spectral and temporal smoothing is applied to the short temporal window plots in order to clearly show the spectral shifts. The color bar is normalized based on the LAPE subtracted data. LAPE-subtracted spectra out to 4 ps are shown in Fig. S5.†

The pump-probe signals from BrU and BrUrd are more clearly observed in the contour plots of the time-resolved photoelectron spectra shown in Fig. 3; the raw data (panels a and d) includes the LAPE signal, and this contribution is subtracted out to 6 eV in panels b and e using the spectrum obtained from 25 mM NaCl aqueous solution with procedure described in Fig. S3.† LAPE subtraction beyond 6 eV was not successful owing to the strong contribution of LAPE from various origins and depletion from the ground state. The LAPE signal from 25 mM NaCl aqueous solution was successfully fitted with a Gaussian function that persists out to eBE ∼4.8 eV and whose width of 38 fs (1*σ*) matches well with the IRF (*σ* = 30 fs) measured in an Ar gas jet (Fig. S4†).^30^ The strong LAPE signal is centered at *t*_0_, whereas the weaker pump-probe signals extend asymmetrically to positive delays. For both BrU and BrUrd, the onsets of the pump-probe signals begin at eBE ∼3 eV, corresponding to excitation to the Franck–Condon (FC) region of the ^1^ππ* state.

**Fig. 4:**
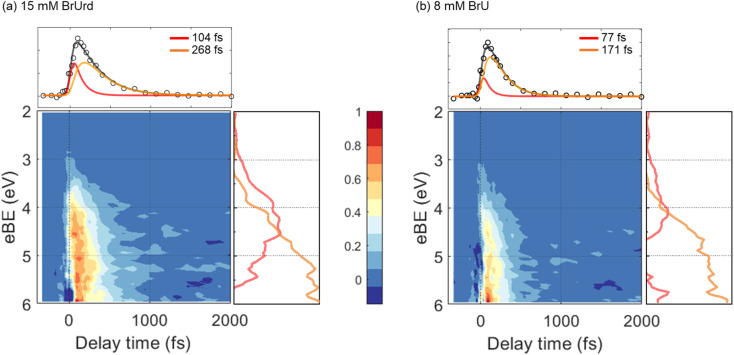
LAPE-subtracted time-resolved photoelectron spectra and associated global lifetime analysis (GLA) results for (a) 15 mM BrUrd and (b) 8 mM BrU aqueous solution. In the upper panels, temporal lineout of GLA results are displayed, while the right panels show evolution-associated spectra (EAS) obtained from GLA. Fast time components resulting from GLA are labeled in red, while slow time components are labeled in blue. The integrated total temporal behavior of the GLA results is depicted by the black solid line in the temporal lineout.

As depicted in Fig. 3(c) and (f), the LAPE-subtracted BrUrd and BrU contour plots exhibit a noticeable spectral shift from lower eBE (3–4.5 eV) to higher eBE (4.5–6 eV) with increasing time delays. More specifically, the initial excited state population of BrUrd around 4.5 eV migrates to higher eBE (4–6 eV) within ∼200 fs and subsequently disappears within a few hundred femtoseconds. Although the photoelectron spectrum of BrU is noisier than that of BrUrd, a similar spectral evolution is also observed in BrU. Fig. 4a also shows a weak signal above 6 eV that grows in over 1.5–2 ps and persists out to 4 ps. As discussed further in Section 4, this signal is attributed to vibrationally hot ground state BrUrd in its ground electronic state.

For quantitative analysis of the excited state relaxation dynamics of BrUrd and BrU, we applied global lifetime analysis (GLA) to both time-resolved spectra to separate spectral components with different time constants. A sequential kinetic model 
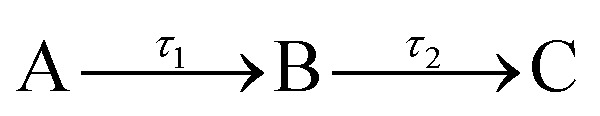
 comprising three states and two time constants was found to reproduce the spectral shift from the low eBE region to high eBE, representing a non-adiabatic transition from the FC-region of the initially-excited ^1^ππ* to a lower-lying electronically excited state. The time-evolving photoelectron spectrum is expressed by eqn (1)1
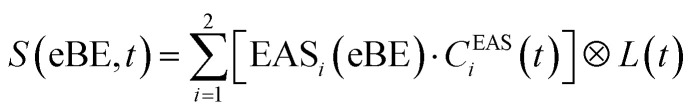


Here, *S*(eBE, *t*) corresponds to the overall 2D data set, EAS_*i*_(eBE) is the evolution-associated spectrum (EAS) representing the photoelectron spectrum of component *i*, and *C*^EAS^_*i*_(*t*) corresponds to the time-dependent intensity of EAS_*i*_, a sum of exponential functions. *L*(*t*) corresponds to an instrumental response function (IRF) taken to be a Gaussian function. The kinetic model applied here describes the initial decay of the FC region (A) to the formation of an intermediate state (B), and subsequent decay to product (C), as follows:

2 *C*_1_^EAS^(*t*) = *a*_1_*e*^−*t*/*τ*_1_^

3 *C*_2_^EAS^(*t*) = *a*_2_(*e*^−*t*/*τ*_2_^ − *e*^−*t*/*τ*_1_^)

Here *τ*_1_ corresponds to the lifetime of the initially excited state, while *τ*_2_ represents decay of the intermediate state signal.

The results of the GLA for BrUrd are shown in Fig. 4a. The EAS_1_ of BrUrd exhibits a fast decay at 3–5 eV with a lifetime of *τ*_1_ = 104 ± 5 fs, accompanied by simultaneous population growth at 3–6 eV. This EAS_1_ adequately describes relaxation from the initially excited state (FC-region of the ^1^ππ* state) to a lower-lying state with a lifetime of *τ*_1_, which effectively reproduces the spectral shift observed in BrUrd within short time delays (<200 fs). The second time component (EAS_2_) was fitted with *τ*_2_ = 268 ± 4 fs. Although signal levels for BrU are slightly lower, a similar EAS result is obtained as for BrUrd (Fig. 4b), with fitted lifetimes *τ*_1_ = 77 ± 3 fs and *τ*_2_ = 171 ± 4 fs. The residual spectra of the GLA are presented in Fig. S6.† We also analyzed the temporal behaviors using a single-exponential decay convoluted with a Gaussian function, but this did not yield a reliable fit for the temporal behavior or accurately simulate the spectral shift as the pump-probe delay increased (Fig. S7†).

## 4. Discussion

### 4.1 Relaxation dynamics of the BrU and BrUrd ππ* state

Our time-resolved LJ-PES experiment elucidates intricate details regarding the dynamics of excited states of BrU and BrUrd in aqueous solution, offering insight into the propagation of wavepackets across multiple electronic states. The high temporal resolution (38 fs) and broad detection range of electron binding energies in our setup yields a comprehensive view of the energy landscape of the excited states. By leveraging these advantages, we propose that the relaxation dynamics of BrU and BrUrd upon UV excitation to the ^1^ππ* state primarily decays to a lower-lying electronically excited state within ∼100 fs by a non-adiabatic transition, before ultimately relaxing to the final photoproducts. This interpretation is strongly supported by the GLA described in Section 3, in which a sequential model involving the initially excited the ^1^ππ* decaying to a short-lived intermediate state with a different EAS captures all of the dynamics out to 1 ps.

Previously, Wang *et al.* observed rapid decay with a lifetime of 0.4 ps for BrUrd in aqueous solution upon 266 nm UV excitation using femtosecond TA experiments,^24^ a somewhat slower value than what is found in the current study. This difference may be attributed to lower temporal resolution (IRF ∼0.3 ps) and limited detection wavelength range in the TA experiment, which may have hindered the differentiation of excited state population transfer to lower-lying states.

To interpret the overall relaxation dynamics of BrU and BrUrd, it is useful to compare the results presented here with those of Thy and related species. Several LJ-PES studies have observed somewhat slower relaxation from the ^1^ππ* excited state of Thy and thymidine compared to what was measured for BrU and BrUrd in this work. Lübcke reported lifetimes of the Thy ^1^ππ* state to be 410 ± 80 fs with a 5.20 eV UV probe photon,^19^ which generally align with LJ-PES experiments using probe photon energies of 6.20 eV by Erickson *et al.*^46^ and 26.4 eV by Miura *et al.*^47^ More importantly, additional decay channels forming the ^1^nπ* state and subsequent decay to the lower-lying triplet state pathways were proposed by Miura *et al.*^47^ with lifetimes measured to be 2.5 ± 0.8 ps for the ^1^nπ* state and >20 ps for the lower-lying triplet state. The Thy studies consistently demonstrate longer lifetimes for the ^1^ππ* state and subsequent decays compared to both BrU and BrUrd in our investigation.

The different time constants for Thy and BrU may stem from differences in the energy ordering of the ^1^nπ* state and ^1^ππ* states. Gas-phase experiments on Thy have proposed that nearly all relaxation pathways proceed by the ^1^nπ* state, while the quantum yield to form the ^1^nπ* state significantly decreases in aqueous solution due to the increased energy gap between the ^1^nπ* and ^1^ππ* states.^18,47^ The situation with the ^1^ππ* and ^1^nπ* states of BrU differs somewhat from Thy. To compare the energetic location of the ^1^ππ* and ^1^nπ* states of BrU in gaseous or aqueous environments, we conducted time-dependent density functional theory (TD-DFT) calculations at the B3LYP/6-311++G(3df,3pd) level of theory. For the calculation in aqueous solution, we adopted the integral equation formalism polarizable continuum model (IEFPCM) with a water dielectric constant (*ε* = 78.39). According to the TD-DFT calculation results (Table 1), the ^1^nπ* state of BrU is located 0.12 eV higher in energy compared to the ^1^ππ* state in the gas phase, and the energy gap between the ^1^nπ* state and ^1^ππ* state increases in aqueous solution to 0.35 eV. These energy gaps are much higher than in Thy, where the ^1^nπ* state is 0.22 eV lower than the ^1^ππ* state in the gas phase and 0.07 eV higher in aqueous solution. Such a large energy gap of BrU in aqueous solution suggests that a non-adiabatic transition to the ^1^nπ* state is unlikely to be the primary relaxation pathway.

**Table 1:** Vertical excitation energies of Thy and BrU in isolated (g) or aqueous (l) environment from TD-DFT calculations with B3LYP/6-311++G(3df,3pd) level of theory with Gaussian16 package. The calculated energy differences between the gas-phase and aqueous solution for each state are shown in parentheses. Note that the character of the S_1_ and S_2_ states switches between gas and liquid phase calculations^a^

Thy	S_1(g)_	S_2(g)_	S_3(g)_	BrU	S_1(g)_	S_2(g)_	S_3(g)_
	^1^nπ*	^1^ππ*	^1^πσ*		^1^ππ*	^1^nπ*	^1^πσ*
	4.75	4.97	5.44		4.60	4.72	4.99
	S_1(aq)_*	S_2(aq)_*	S_3(aq)_*		S_1(aq)_*	S_2(aq)_*	S_3(aq)_*
	^1^ππ*	^1^nπ*	^1^πσ*		^1^ππ*	^1^nπ*	^1^πσ*
	4.92(-0.05)	4.98 (+0.23)	5.80 (+0.36)		4.61(+0.01)	4.96 (+0.24)	5.06 (+0.07)

a * IEFPCM solvation model was applied in order to account for solvent interaction.

### 4.2 Possible role of the ^1^πσ* state and C–Br bond dissociation

**Fig. 5:**
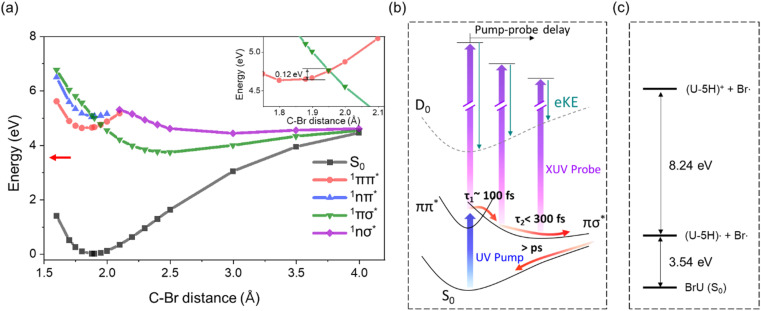
(a) Potential energy curves of S_0_ (black), ^1^ππ* (red), ^1^nπ* (blue), ^1^πσ* (green), and ^1^nσ* (purple) of BrU along the C–Br elongation coordinate calculated by TD-DFT at the B3LYP/6-311++G(3df,3pd) level of theory under integral equation formalism polarizable continuum model (IEFPCM) solvation model in water. The crossing point between ^1^ππ* and ^1^πσ* is enlarged in the inset. Asymptotic dissociation energy for geometry optimized uracil-5-yl radical and bromine atom (3.54 eV) is shown in red arrow. (b) Schematic potential energy diagram and the proposed relaxation pathway of BrU and BrUrd following C–Br elongation coordinate in the “effective ionization window” (2–6 eV). (c) Energy diagram of the asymptotic region of C–Br dissociation for uracil-5-yl (U-5H) radical and bromine atom fragments. The diagram is based on calculations using the B3LYP/6-311++G(3df,3pd) level of theory and the IEFPCM solvation model in water.

The shorter lifetime observed for the ^1^ππ* state in BrU (and BrUd) compared to Thy and the bi-exponential behavior suggest consideration of the dissociative ^1^πσ* state in the relaxation dynamics as the intermediate state for which the signal decays on a 200 fs time scale. TD-DFT calculations (Table 1) for BrU in aqueous solution reveal a ^1^πσ* state positioned in close proximity to the ^1^nπ* state and lying ∼0.45 eV above the ^1^ππ* state. While this energy gap appears substantial, the two states may intersect at elongated C–Br geometries. To verify this, we conducted a rigid scan using TD-DFT calculations for BrU along the C–Br bond elongation coordinate. As depicted in Fig. 5, the potential energy curve for the ^1^πσ* state rapidly descends at longer C–Br distances from the FC-region, intersecting the potential energy curve for the ^1^ππ* state. The crossing point lies only 0.12 eV above the S_1_ FC-region. The small energy gap between the S_1_ minimum and the ^1^ππ*/^1^πσ* crossing point suggests the existence of an IC pathway to the ^1^πσ* state, potentially leading to C–Br bond dissociation. The asymptotic energy for the uracil-5-yl (U-5H) radical and bromine atom fragments is calculated to be 3.54 eV, significantly lower than the S_1_ minimum energy and the ^1^ππ*/^1^πσ* crossing point. Note that the asymptotic energy is calculated for the optimized structure of U-5H radical product, whereas the potential energy curve, obtained from a rigid scan, predicts higher asymptotic energy.

Attribution of the ^1^πσ* state as the intermediate is consistent with the EAS for this state, shown as orange curves in Fig. 4. These spectra peak at 6 eV, and are quite different from those for the ^1^ππ* state. Since the ^1^πσ* state is repulsive out to the dissociation limit, one expects rapid elongation of the C–Br bond length on this state. Fig. 5b shows a schematic of the potential energy curve for *D*_0_, the cation ground state that correlates to (U-5H)^+^ + Br products. Our calculated ionization energy for the (U-5H) radical is 8.24 eV (see Fig. 5c), which is higher than that of BrU (6.88 eV). As a result, the vertical ionization energy of the ^1^πσ* state increases with C–Br bond length. Hence, the strong contribution to the EAS at high eBE is consistent with wavepacket propagation on the ^1^πσ* state.

However, the 200 fs decay of the signal from the ^1^πσ* state does not represent the lifetime of this state. Instead, it reflects the “effective ionization window” for dynamics on this state over the energy covered by the GLA, *i.e.* out to 6 eV. Although the 21.7 eV probe pulse can ionize the ^1^πσ* anywhere along the C–Br reaction coordinate, the increase in VIE with C–Br bond length causes signal from the ^1^πσ* state to overlap with the much stronger photoelectron signal from ground state BrU and BrUrd, which starts around 6 eV (insets of Fig. 1). Hence, elucidating excited state dynamics beyond 6 eV is unreliable.

Although relaxation through the ^1^πσ* is energetically viable, not all the population in the ^1^ππ* state necessarily undergoes C–Br bond dissociation. Previous studies on UV irradiation of BrU in aqueous solution have reported C–Br homolysis with a quantum yield of less than ∼5%,^48–51^ depending on solution types and concentrations. In aqueous solution, Hutchinson *et al.* reported a C–Br homolysis quantum yield of *Φ* = 0.002 for BrU, determined by measuring the formation of the uracil-5-yl radical upon 254 nm irradiation using chromatography.^52^ This result is consistent with a later study by Campbell *et al.* that reported a quantum yield of *Φ* = 0.0018 based on the disappearance of BrU.^48^

These earlier studies would be consistent with the dynamics on the πσ* state inferred from our experiment if dissociation were inhibited by solvent caging effects.^53–55^ This leads to the question of whether our experiments can provide independent verification of the dissociation yield. Ideally, one would measure production of the U-5H radical and/or the Br fragment, but the VIEs of both species are too high to distinguish from ground state solute and solvent photoelectron signal; the gas phase VIE of Br is 11.8 eV. However, we observe a weak recovery of photoelectron signal around 6 eV for BrUrd emerging at ∼2 ps, which may indicate the formation of the hot S_0_ ground state (Fig. S8†). This feature overlaps with the noise background from the strong water signals and the depletion of target molecule's ground state, making it challenging to analyze its temporal behavior. Nevertheless, the recovery of signal in this eBE range at longer time delays, compared to the measured decay components (<300 fs) associated with excited state dynamics, suggests re-generation of the reactant *via* solvent caging effects. The ∼2 ps emergence time of this spectral feature aligns with previous measurements of geminate recombination of I_2_ in non-polar solvents^56,57^ or I_3_^−^ in aqueous solution,^58,59^ In any case, while the current configuration of our experiment is insensitive to the fragmentation products of photoexcited BrU and BrUrd, it provides some evidence for the re-generation of BrUrd *via* geminate recombination, consistent with previous reports of a low dissociation quantum yield. The absence of the recovery for BrU is likely due to the low concentration and the stronger ground state depletion feature. We plan to extend our photon energy range out to 90 eV (or higher) in the near future, which will enable one to eject Br(3d) core electrons in a less congested region of the photoelectron spectrum and thus provide more insight into the extent to which dissociation of these species occurs in solution.

### 4.3 BrU and BrUrd as a radiosensitizer by photolysis

Several studies have highlighted the role of BrU and BrUrd as radiosensitizers, emphasizing DEA as a mechanism more significant than direct photoexcitation to electronic excited state followed by photolysis.^21,22,60–63^ Notably, Wang *et al.*^24^ observed ultrafast electron transfer reaction of BrUrd initiated by photoionization of precursor water to form prehydrated electrons (e_pre_^−^). In their investigation, the electron transfer reaction, initiated by two-photon ionization of water solvent with a 320 nm UV pump, occurred within ∼0.2 ps, leading to the decay of the BrdU*^−^ transition state within ∼1.5 ps to yield Br^−^ and radical dU* fragments. Attempts with a 266 nm UV pump to directly excite BrUrd to the ^1^ππ* state in their work did not show evidence for formation of Br atoms or uridin-5-yl radicals. Also, an ultrafast electron transfer from deoxyadenosine monophosphate (dAMP) to BrUrd promoted by e_pre_^−^ attachment to dAMP was suggested to yield reactive uridinyl radical by the same group.^64^ The indirect pathway based on DEA was found to be effective in promoting the radiosensitizing effect of BrUrd compared to direct photoexcitation to neutral excited states.

Previous gas-phase DFT calculations performed by Li *et al.* suggested that the lowest anionic state of BrU^−^, characterized by a mixed nature of two anionic states (π*(A′′) and σ*(A′)),^60^ is responsible for the DEA process leading to C–Br fragmentation, overcoming a potential barrier of 1.88 kcal mol^−1^ (0.08 eV). More recently, Cornetta *et al.* employed a semiclassical *ab initio* calculation to account for short-lived transient negative anionic states (shape resonances) above the neutral ground state.^63^ They suggested that slow electron attachment to the π* anion shape resonance predominantly facilitates efficient coupling (within 100 fs) to the σ*(C–Br) state, resulting in Br^−^ fragment anion formation. It is important to note that the mixed π*(A′′) – σ*(A′) character of BrU^−^ represents the lowest valence anionic state, thereby directing most of the reaction flux after electron attachment through the dissociative σ*(C–Br) surface, unlike the relaxation dynamics in the neutral BrU proposed here.

In neutral BrU, efficient internal conversion to the S_0_ ground state combined with solvent caging inhibits C–Br dissociation. However, once an electron attaches, Br^−^ formation is very facile through coupling to the dissociative σ*(C–Br) surface. Although most theoretical/experimental studies on DEA are in the gas-phase,^12,61,62,65^ the characteristics of BrU^−^ potential energy surfaces could be relevant to the DEA process involving pre-hydrated electrons in aqueous solutions. Several experimental observations on DNA sequences modified with BrU have demonstrated a strong correlation with pre-hydrated electrons generated by UV photon^64^ or low-energy electron irradiation,^66,67^ highlighting the role of BrU (and BrUrd) as a radiosensitizer in the context of anion chemistry.

## 5. Conclusions

Ultrafast photo-relaxation dynamics of BrU and BrUrd in an aqueous environment were investigated using femtosecond time-resolved photoelectron spectroscopy in liquid flat jets. Employing XUV probes with 21.7 eV photons enabled differentiation and tracking of overall relaxation dynamics involving a lower-lying intermediate state. Upon 4.66 eV UV-induced photoexcitation to the first singlet excited state (^1^ππ*), both species exhibited ultrafast relaxation to lower-lying state, accompanied by a spectral shift to higher electron binding energies. GLA results revealed the ultrafast decay of the initial ^1^ππ* state at eBE of 3–4.5 eV with the lifetimes of *τ*_1_ = 124 fs and 77 fs for BrUrd and BrU, respectively, and subsequent formation of a lower-lying electronically excited state, primarily relaxing towards the S_0_ ground state.

TD-DFT calculations suggest that the lower-lying intermediate state is the repulsive ^1^πσ* state, intersecting the ^1^ππ* state at geometries at elongated C–Br bond lengths. Although relaxation through the ^1^πσ* state appears to be the main decay pathway, the primary product channel is anticipated to be the S_0_ ground state *via* geminate recombination based on the previously measured low quantum yield for C–Br bond homolysis, a result attributed here to solvent caging. This work raises the possibility that even though UV excitation populates the repulsive ^1^πσ* state, direct UV excitation of BrU and BrUrd may have a limited role in their photosensitizing ability. Instead, an indirect pathway involving electron transfer reactions from the solvent network could be more efficient, occurring within the framework of the anion *via* dissociative electron attachment.

## Data availability

The data sets in this study are available from the corresponding author on request.

## Author contributions

Do Hyung Kang: data curation (lead), investigation (lead), methodology (equal), visualization (lead), writing – original draft preparation (lead), writing – review & editing (equal). Masafumi Koga: data curation (equal), investigation (equal), methodology (lead), writing – original draft preparation (supporting), writing – review & editing (supporting). Neal Haldar: investigation (supporting), data curation (supporting). Daniel M. Neumark: conceptualization (lead), project administration (lead), resources (lead), supervision (lead), validation (lead), writing – review & editing (equal).

## Conflicts of interest

There are no conflicts of interest to declare.

## Supplementary Material

SC-OLF-D4SC03920C-s001
