# Supplementary material for: Dynamics of photoexcited 5-bromouracil and 5-bromo-2′-deoxyuridine studied by extreme ultraviolet time-resolved photoelectron spectroscopy in liquid flat jets
Source: Chem Sci. 2024 Sep 26;15(41):17245–55. doi: 10.1039/d4sc03920c (PMC11446316; doi:10.1039/d4sc03920c)
Supplement: SC-015-D4SC03920C-s001 [file SC-015-D4SC03920C-s001.pdf]

Supplementary Information (SI) for Chemical Science.
This journal is © The Royal Society of Chemistry 2024

Supplementary Information for
Dynamics of Photoexcited 5-Bromouracil and 5-Bromo-2'-Deoxyuridine Studied by Extreme Ultraviolet Time-resolved Photoelectron Spectroscopy in Liquid Flat Jets

Do Hyung Kang[1], Masafumi Koga[1], Neal Haldar[1], and Daniel M Neumark[1,2*]

[1] *Department of Chemistry, University of California, Berkeley, California 94720, USA*

[2] *Chemical Science Division, Lawrence Berkeley National Laboratory, Berkeley, California 94720, USA*

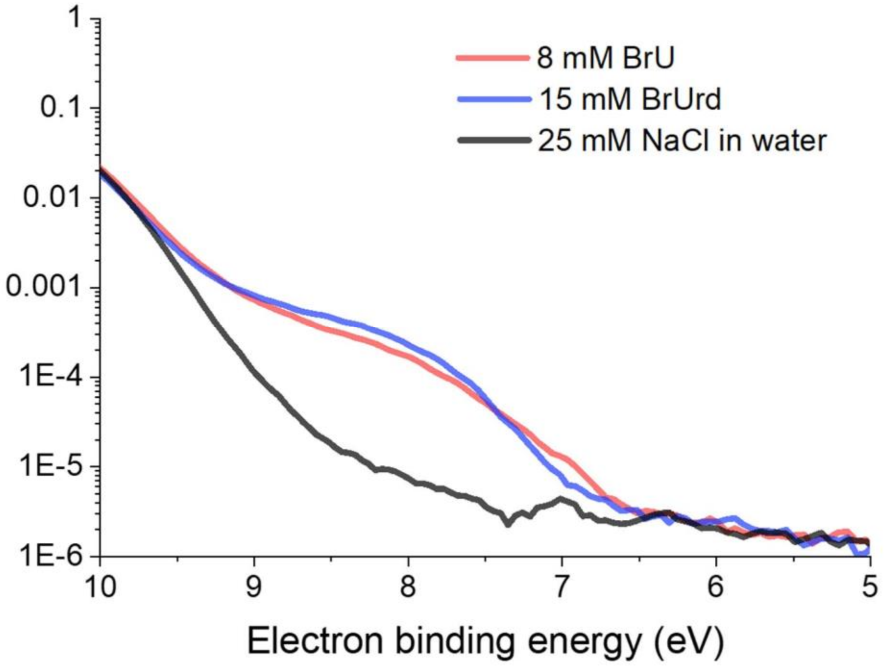

Fig. S1 XUV photoelectron spectra of (a) 8 mM BrU, (b) 15 mM BrUrd, and (c) 25 mM NaCl aqueous solution in fla t liquid jets.

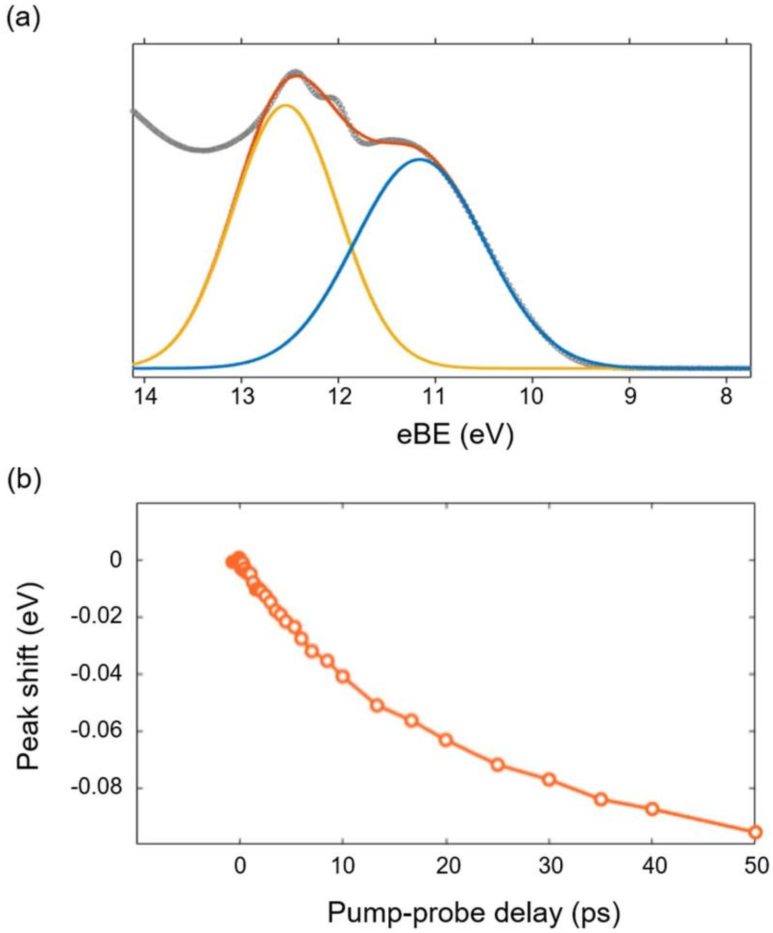

Fig. S2 Space-charge correction of the liquid water peak for 15 mM BrUrd aqueous solution with a 500 u W UV pump. (a) Photoelectron spectrum deconvoluted with two Gaussian functions, corresponding to (yellow) $1b_{1(g)}$ and (blue) $1b_{1(l)}$ peaks. The gray circles depict the photoelectron data points, while the red solid line represents the total fit achieved with the two Gaussian functions. (b) Profile of the space charge induced shift of the $1b_{1(l)}$ water peak depending on the temporal delays between UV pump – XUV probe.

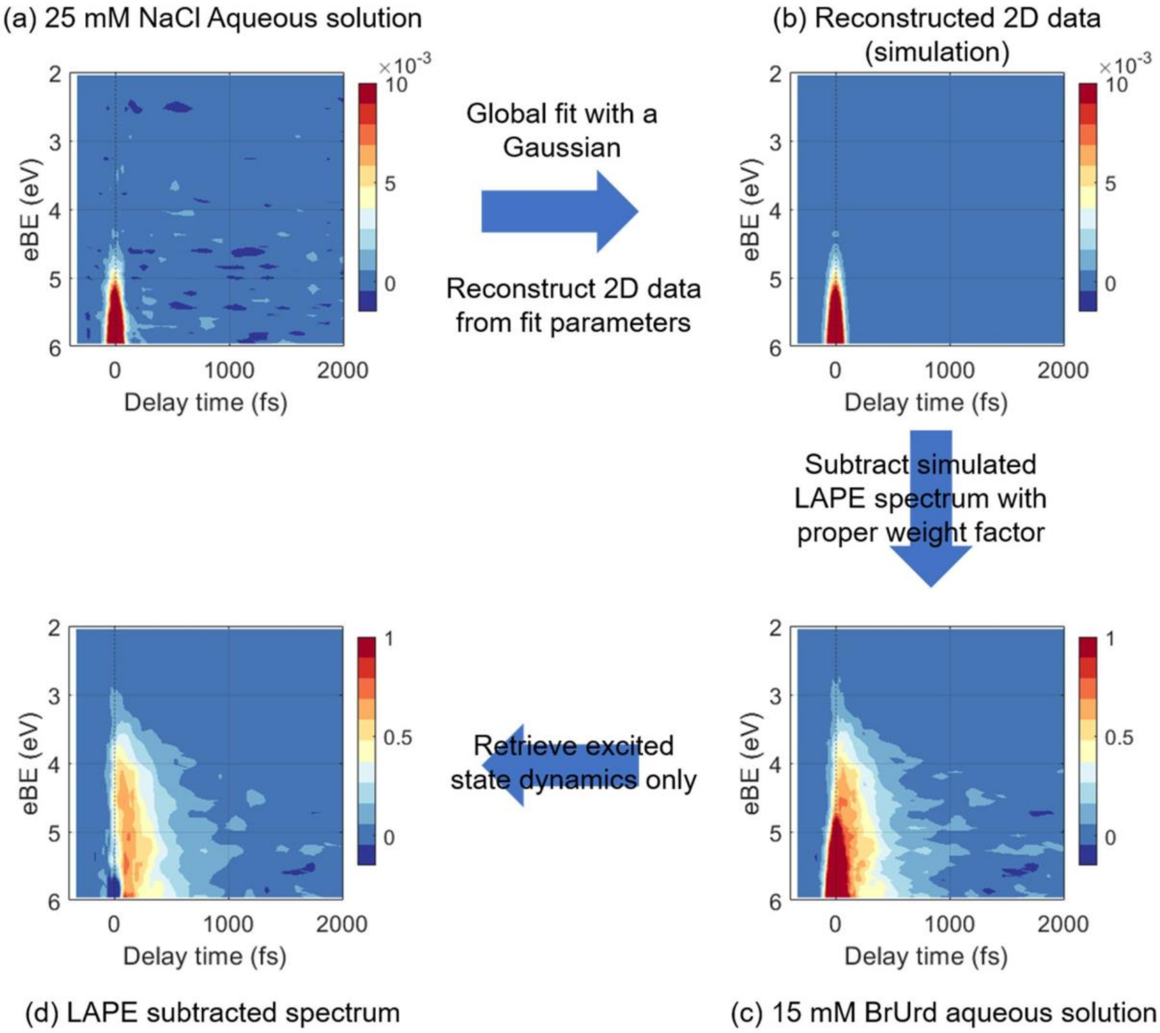

Fig. S3 Schematic description of the laser-assisted photoelectric effect (LAPE) signal subtraction method. (a) The LAPE-only spectrum was obtained from a 25 mM NaCl aqueous solution and then globally fitted with a Gaussian function to reconstruct (b) the simulated 2D data. The LAPE spectrum is then subtracted from the (c) spectrum acquired from the target solution using appropriate weight factors, resulting in the (d) LAPE-subtracted spectrum.

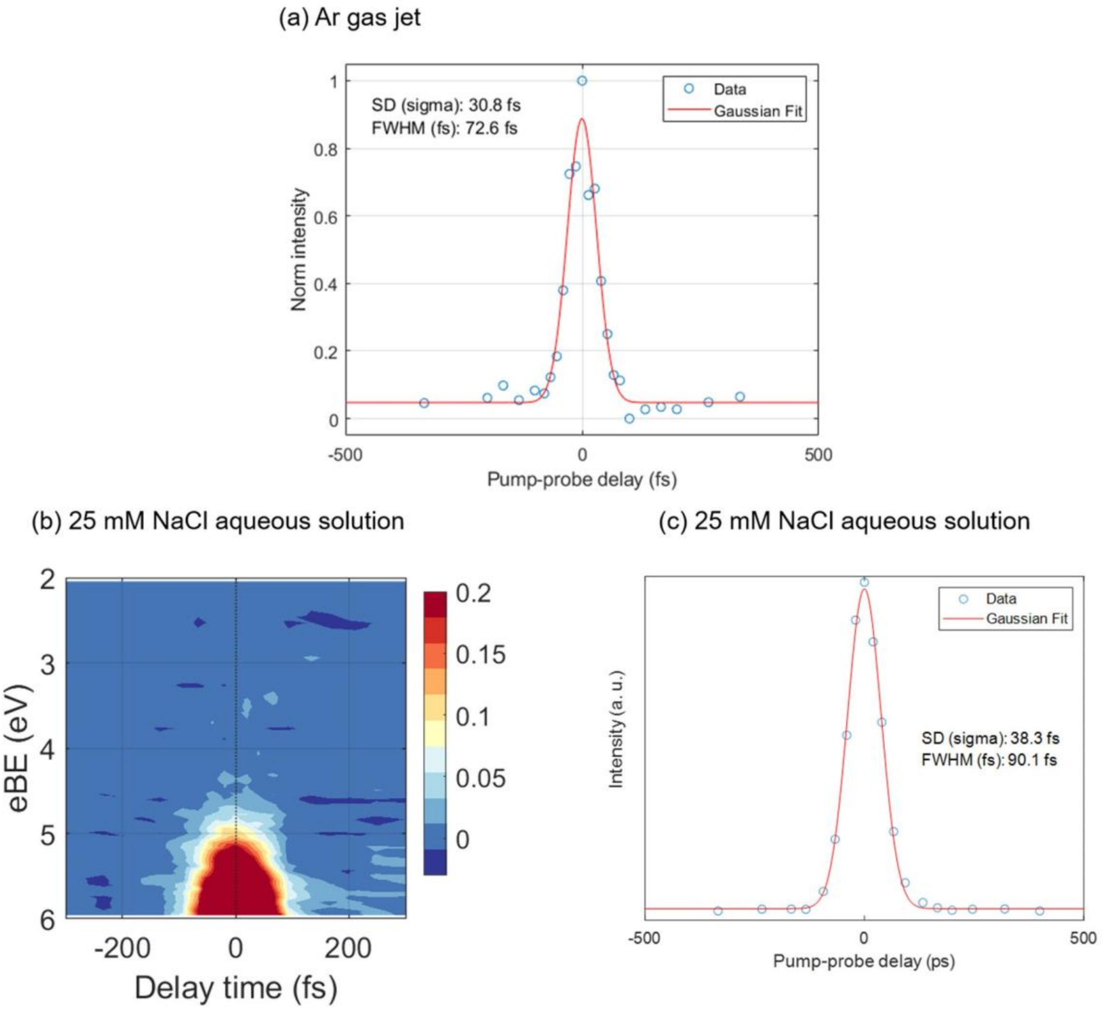

Fig. S4 Cross-correlation of a 4.66 eV UV pump and a 21.7 eV XUV probe facilitated by LAPE in (a) Ar gas jet and (b-c) a 25 mM NaCl aqueous solution in a liquid jet. Temporal lineouts for both (a) Ar gas and (c) liquid jets are generated by integrating the photoelectron signals in 14 – 15 eV electron kinetic energy region. The instrumen t response function (IRF) is determined by fitting the temporal lineout with a Gaussian function. The insets display the standard deviation (1 σ) and the full-width at half-maximum (FWHM) values from the fit.

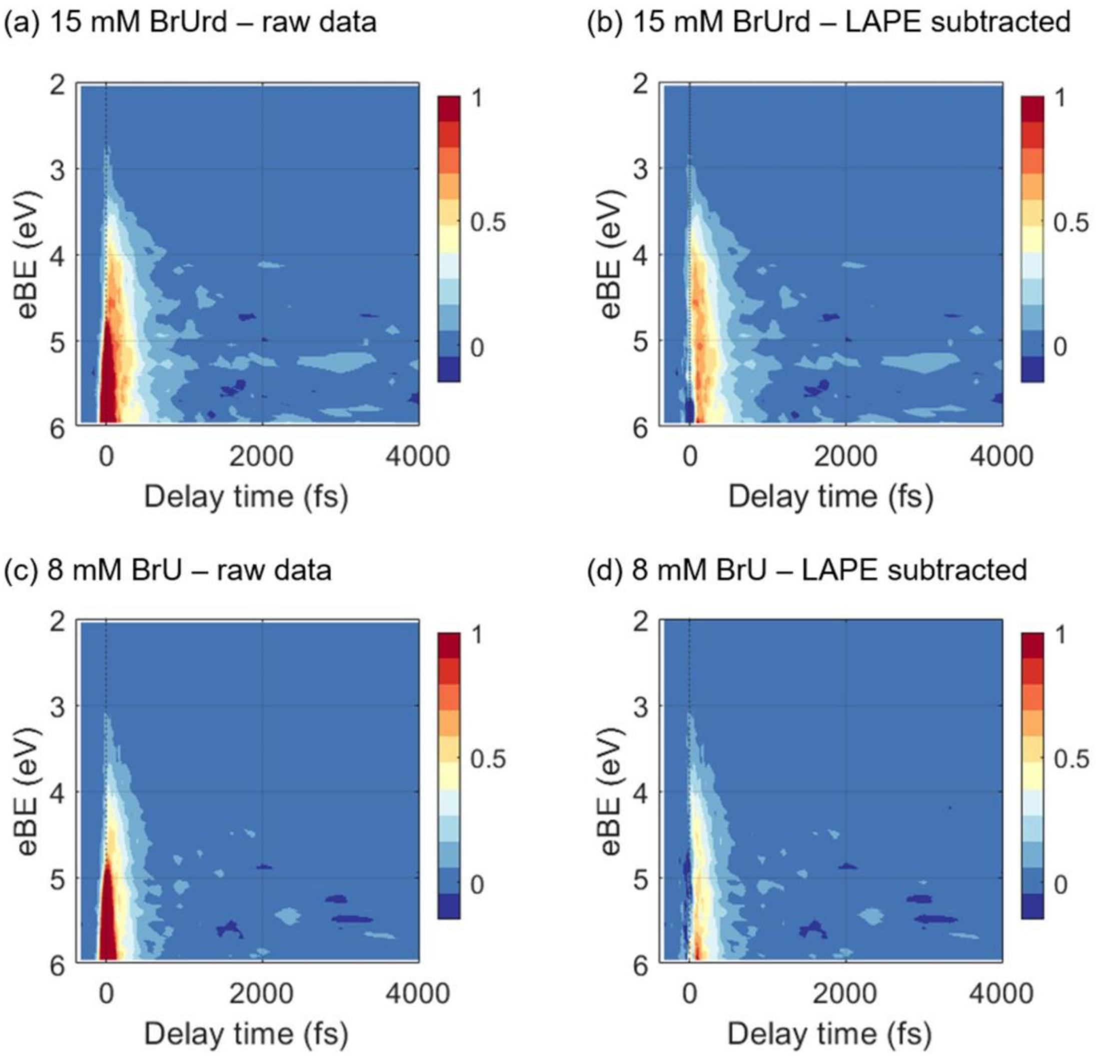

Fig. S5 Full-range 2D plots of time-resolved photoelectron spectra for (a-b) 15 mM BrUrd and (c-d) 8 mM BrU up to 4 ps temporal window.

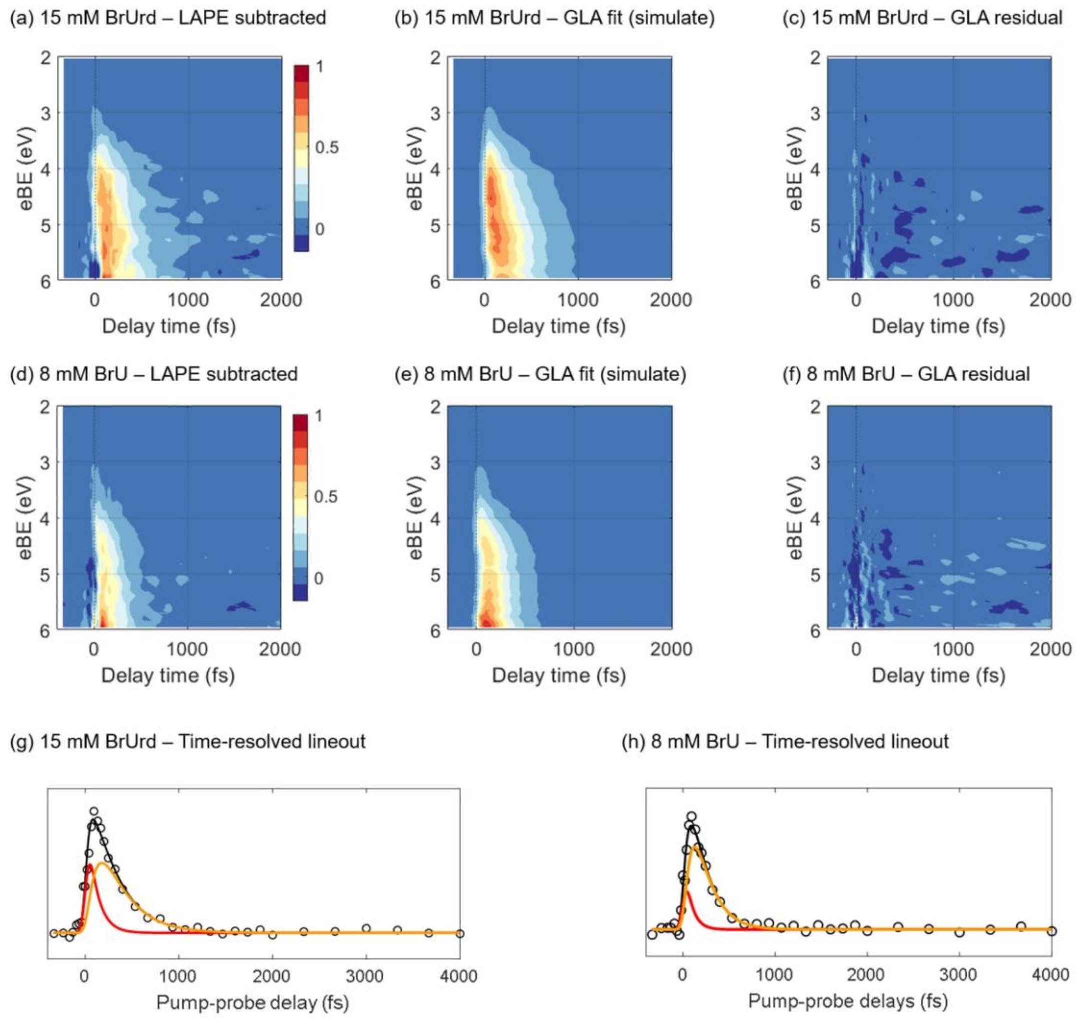

Fig. S6 LAPE-subtracted contour plots and GLA results of (a-c) 15 mM BrUrd and (d-f) 8 mM BrU aqueous solution. The (b and e) reconstructed 2D plots from GLA fit and (c and f) residuals of the evolution-associated spectra (EAS) fit for both spectra are depicted. Full temporal range of time-resolved lineouts for (g) 15 mM BrUrd and (h) 8 mM BrrU aqueous solutions. 3 points spectral average is applied on the LAPE subtracted data.

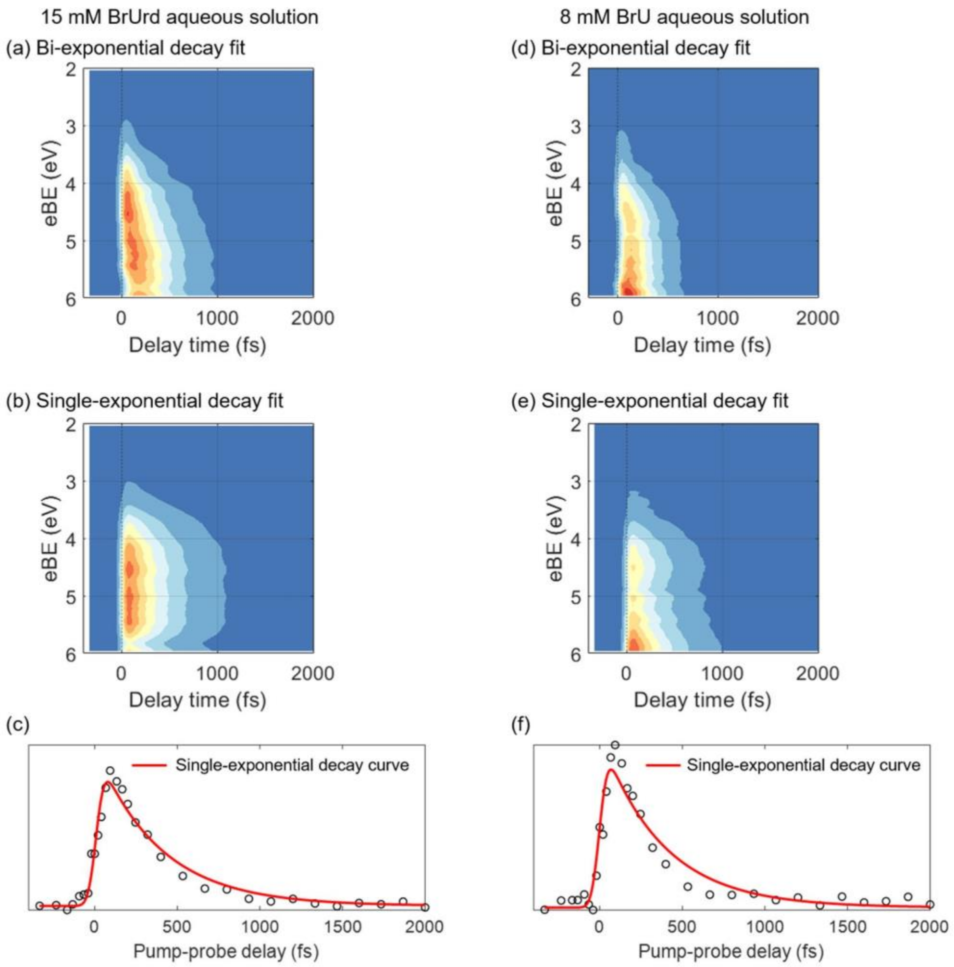

Fig. S7 Comparison between (a and d) bi-exponential (stepwise) decay fit and (b and e) single-exponential decay fit for (a-c) 15 mM BrUrd and (d-f) 8 mM BrU aqueous solution. (c and f) Temporal lineouts for total integrated photoelectron signal (black open circle) and single-exponential decay fit (red solid line), which exhibits significant mismatches.

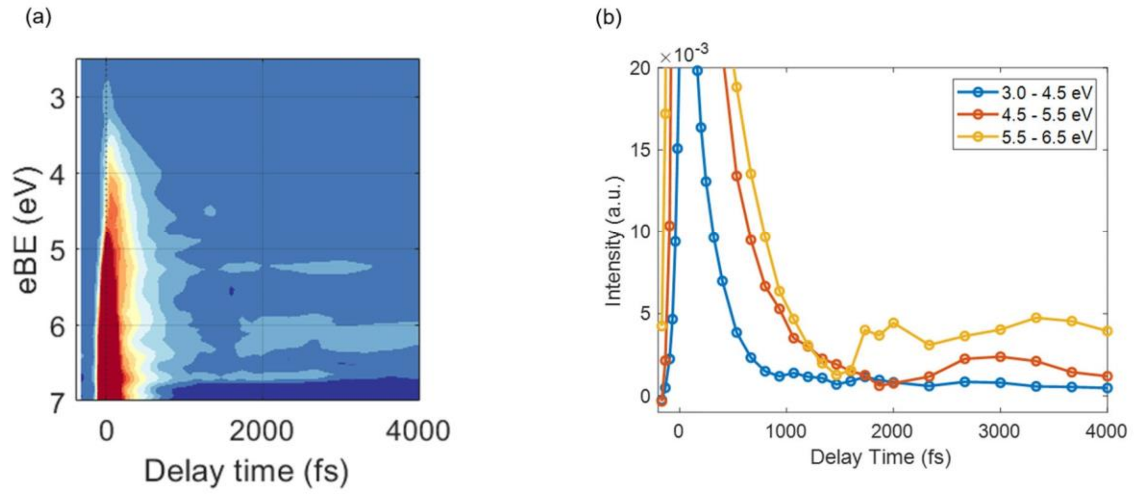

Fig. S8 (a) Contour plots of time-resolved photoelectron spectra of BrUrd, with 3 points spectral and temporal smoothing. Note the strong depletion feature around 7 eV, corresponding to ground state bleach, and the rise in signal observed from ~2 ps at ~6 eV. (b) Time-resolved lineouts from different spectral ranges. The feature emerging around ~2 ps is observable in the 5.5–6.5 eV spectral region.
